# Magnetoencephalography Hyperscanning Evidence of Differing Cognitive Strategies Due to Social Role During Auditory Communication

**DOI:** 10.3389/fnins.2022.790057

**Published:** 2022-08-02

**Authors:** Nano Yoneta, Hayato Watanabe, Atsushi Shimojo, Kazuyoshi Takano, Takuya Saito, Kazuyori Yagyu, Hideaki Shiraishi, Koichi Yokosawa, Jared Boasen

**Affiliations:** ^1^Graduate School of Health Sciences, Hokkaido University, Sapporo, Japan; ^2^Faculty of Health Sciences, Hokkaido University, Sapporo, Japan; ^3^Department of Child Studies, Toyooka Junior College, Toyooka, Japan; ^4^Department of Child and Adolescent Psychiatry, Hokkaido University Hospital, Sapporo, Japan; ^5^Department of Pediatrics, Hokkaido University Hospital, Sapporo, Japan; ^6^Tech3Lab, HEC Montréal, Montréal, QC, Canada

**Keywords:** communication, improvisation, social, music, hyperscanning, MEG

## Abstract

Auditory communication is an essential form of human social interaction. However, the intra-brain cortical-oscillatory drivers of auditory communication exchange remain relatively unexplored. We used improvisational music performance to simulate and capture the creativity and turn-taking dynamics of natural auditory communication. Using magnetoencephalography (MEG) hyperscanning in musicians, we targeted brain activity during periods of music communication imagery, and separately analyzed theta (5–7 Hz), alpha (8–13 Hz), and beta (15–29 Hz) source-level activity using a within-subjects, two-factor approach which considered the assigned social role of the subject (leader or follower) and whether communication responses were improvisational (yes or no). Theta activity related to improvisational communication and social role significantly interacted in the left isthmus cingulate cortex. Social role was furthermore differentiated by pronounced occipital alpha and beta amplitude increases suggestive of working memory retention engagement in Followers but not Leaders. The results offer compelling evidence for both musical and social neuroscience that the cognitive strategies, and correspondingly the memory and attention-associated oscillatory brain activities of interlocutors during communication differs according to their social role/hierarchy, thereby indicating that social role/hierarchy needs to be controlled for in social neuroscience research.

## Introduction

Auditory communication, or communication based on sound, is one of the most important forms of social interaction we use in our daily lives. Verbal or non-verbal, oral or non-oral, auditory communication is often dynamic and creative and depends upon sensitivity and spontaneous responsivity not only to detailed characteristics of sound, but also to social context, hierarchy and roles (Berry et al., 1997; Dijksterhuis and Bargh, 2001). Understanding the neurophysiological mechanisms underlying auditory communication is an important target of social neuroscience (see review by Liu et al., 2018). Historically, neuroimaging investigations regarding social-cognitive processing have generally employed passive designs such as viewing images or listening to audio (see review by Hari and Kujala, 2009). However, there has been a shift over the past 10 years toward investigations of brain activities in two (or more) people during real-time ecologically valid social interactions using a neuroimaging technique called hyperscanning (Hari et al., 2015; Redcay and Schilbach, 2019).

Many hyperscanning studies of social interactions involving auditory communication have been concerned with the synchrony within or between brains during cooperative or joint action (for reviews, see Keller et al., 2014; Bieńkiewicz et al., 2021; Kelsen et al., 2022). For instance, Konvalinka et al. (2014) used electroencephalographic (EEG) hyperscanning to examine alpha (8–12 Hz) and beta (13–30 Hz) activity during auditory interactions in the form of index finger tapping. However, with only a single sound used and a goal of synchronizing tapping behavior rather than exchanging unique information, the design did not capture the improvisational freedom of natural communication exchange. A design which featured more complex and natural auditory communication was implemented by Novembre et al. (2016) and Gugnowska et al. (2022), who respectively investigated inter-brain synchrony and intra brain EEG neurocorrelates in delta (1–3 Hz), theta (4–7 Hz), alpha (8–12 Hz), beta (13–30 Hz), and gamma (30–40 Hz) frequency bands during joint piano playing. However, these studies used fixed musical scores for both subjects, and did not employ turn-taking, and thus lacked the creativity and dynamics of natural two-way communication. Similar limitations also apply to a study by Sänger et al. (2012), who used EEG hyperscanning to target delta (1–4 Hz) and theta (4–8 Hz) inter-brain synchrony during guitar playing. A follow-up study to Sänger et al. (2012) by Müller et al. (2013) addressed the limitation of creativity by employing joint improvisational guitar playing. However, if one wishes to investigate the neurocorrelates of auditory communication, not social cooperation, then a turn-taking paradigm is arguably more appropriate.

Hyperscanning studies employing turn-taking paradigms of auditory communication have been reported, although some are still limited with respect to the creativity and dynamism of the communication. For example, Kawasaki et al. (2013) used EEG hyperscanning in a constrained communication paradigm where dyads (human-to-human or human-to-machine) took turns saying the letters of the alphabet in order from A to G. They reported higher inter brain synchronization of theta/alpha (6–12 Hz) activity at left temporoparietal electrodes between dyad members when they directly interacted vs. when one observed the other interacting with a machine. They attempted to attribute these results to working memory function and social cognition. Pan et al. (2018) used a more ecological design with functional near-infrared spectroscopy (fNIRS) to investigate the relationship between IBS and song learning in teacher–student dyads, but the singing-based communication was scripted. They reported greater inter brain synchrony bilaterally at measurement sites over the inferior frontal cortex during interactive learning compared to non-interactive learning. These findings were attributed to the role of the IFC in language processing as well as the potential involvement of the mirror neuron system, which has been proposed to facilitate social interactions (Gallese, 2013). Meanwhile, highly ecologically valid designs of auditory communication exchanges have also been reported. One study by Jiang et al. (2012) used fNIRS to investigate inter-brain synchrony during communication between dyads using a two-by-two design where communication was either face-to-face or back-to-back and either open dialogue or monologue. Although their sensor array was limited to 20 measurement channels over the left hemisphere, their analyses indicated that increased inter-brain synchronization at the T3 position was positively associated with interpersonal behaviors such as turn-taking involved in communication, a result they also attributed to mirror neuron system involvement. In another highly ecologically valid design, also using fNIRS, Zhang et al. (2018) observed that during open communication between counselors and patients, right temporoparietal IBS was positively associated with the seriousness of the discourse in association with higher measures of self-reported alliance between dyad members. They related their findings to reports of the role of the temporoparietal junction in social connectedness and cognitive empathy (Atique et al., 2011; Kinreich et al., 2017). In short, these studies support that brain activity recorded *via* hyperscanning during auditory communication exchanges can be differentiated according to the freedom and depth of the interpersonal interactions involved in the communication. However, as Pan et al. (2018) observed, they all suffer from the same potential limitation that the inter brain synchronization differences are merely due to the two brains simultaneously processing the same sensory information. Moreover, the functional explanations of the results also remain tenuous because the analyses do not identify neurophysiological changes at the single brain level which are associated with the cognitive factors involved in the communication. Thus, identifying the intracortical spectro-spatial drivers of the differential factors of auditory communication (e.g., free vs. constrained expression) remains an important gap in research on auditory communication that needs to be filled. To this end, magnetoencephalography, which has excellent temporal and spatial resolution, could constructively contribute. Granted, the neuroimaging modality of magnetoencephalography (MEG) is far more sensitive to movement than other modalities such as fNIRS. However, insight toward an appropriate communication paradigm can be drawn from the aforementioned examples and from musical neuroscience.

Indeed, in parallel to hyperscanning research, there has been a rapid expansion of musical improvisation neuroscience research over the past 10 years across numerous neuroimaging domains (Bashwiner et al., 2020). In many ways, musical improvisation is ideally suited for neuroimaging studies of auditory communication because the temporal length of auditory exchange can be easily fixed using a set tempo, while the content of the auditory exchange can be created freely. This intrinsically results in epochs of identical length that can easily be compared behaviorally and neurophysiologically between conditions. This is precisely the design strategy used by Donnay et al. (2014) in fMRI and emulated by Boasen et al. (2018) with MEG where the subjects being scanned performed musical exchanges with either a person outside the scanner or with prerecorded musical phrases, respectively. Donnay et al. (2014) interpreted their findings through the lens of language and communication, proposing that the widespread brain activations they observed during improvisational exchanges were supportive that musical communication operates *via* a syntactic processing network similarly to language. Boasen et al. (2018) used an analytical approach which targeted oscillatory amplitude envelope changes in the beta (15–29 Hz), alpha (8–12 Hz), and theta (5–7 Hz) frequency bands, and corroborated the importance of the prefrontal, parietal, and temporal cortices, respectively, for differentiating brain activity during improvisational exchanges. Together, these studies offer important insight into the neurocorrelates underlying the creative and emergent nature of auditory communication. However, they fall short in their ability to directly neurophysiologically capture the social dynamics involved in the exchange, and how social roles (leader and follower) might affect the brain activities underlying auditory communication exchange.

Taking social roles into account is important because humans are known to attune their phonology, wording, and patterning of speech communication in association with the social hierarchy of the person with whom they are speaking (Carrier, 1999; Kacewicz et al., 2014). Additionally, humans exhibit greater gaze-cuing behavior or attentiveness toward faces associated with higher perceived socioeconomic status (Dalmaso et al., 2012) or those regarded as leaders rather than followers (Capozzi et al., 2016). Neurophysiologically, sensitivity to the social status of others has been differentiated by brain activity in prefrontal and right parietal areas (see review by Mattan et al., 2017). For instance, subjects exhibited greater activation of occipital/parietal and right prefrontal cortices when viewing fictitious gamers who were perceived as having a higher vs. lower skill level (Zink et al., 2008). Meanwhile, brain activation in left occipital, right temporoparietal, and right ventromedial prefrontal areas were observed to significantly interact according to the perception of financial and moral status type and the perception of status level (Cloutier and Gyurovski, 2013). Particularly the involvement of right frontoparietal areas in perception of social status is a recognized phenomenon in social neuroscience which has been attributed to the frontoparietal attentional network, where top-down activation of the prefrontal cortex is thought to mediate attention-related brain activity (Corbetta and Shulman, 2002; Corbetta et al., 2008; Levy and Wagner, 2011). However, prefrontal activity appears to be important for differentiating not only the perception of social status but also social role during a social interaction. Indeed, in their aforementioned synchronized finger tapping study, Konvalinka et al. (2014) observed spontaneous emergence of leader/follower roles which were differentiated by significantly stronger frontal alpha (10 Hz) desynchronization in leaders than followers during task anticipation and execution. Meanwhile, Vanzella et al. (2019), who used fNIRS to study brain activation changes associated with social role in violin duets, observed higher right temporoparietal activity when subjects played the role of second compared to first violin. Thus, even during non-verbal auditory communication, humans will adopt differing social hierarchies to facilitate the communication, and these hierarchies can be differentiated by brain activity. Therefore, it is crucial that any investigation of brain activity during human communication, verbal or otherwise, either controls or accounts for the social hierarchies/roles of the subjects in the experimental design and analytical models.

Another aspect of experimental design that is very important to acknowledge is that the physical movement involved in communication can introduce noise into the brain activity recording (Gross et al., 2013). Dealing with physical movement artifacts is particularly a concern for MEG recordings as the sensors are not attached to the head of the subject, and complete immobilization of the head is not always possible (Larson and Taulu, 2017). One strategy to avoid these issues associated with physical movement when motor-related brain activity is the target is to record brain activity during mental imagery of the motor-related task of interest (Boasen et al., 2018). Numerous causal network connections are reportedly shared between brain activity during mental imagery of musical performance and that during physical performance (Adhikari et al., 2016). Moreover, brain activity during mental imagery of music performance has been shown to modulate according to targeted beat and meter frequency (Okawa et al., 2017), and correlate to source-level modulation during actual listening to the imagined music (Sanyal et al., 2016; Martin et al., 2017; Zhang et al., 2017). Meanwhile, the location of cortical activation patterns during speech imagery, such as the left Brocca’s and Wernike’s area and the superior temporal cortex, are also well known to overlap with that of actual speech, and brain computer interface researchers are actively pursuing decoding semantic content from encephalographic activity recorded during imagined speech (Rabbani et al., 2019). This evidence collectively indicates that brain activity recorded during mental imagery of both musical performance and verbal communication shares numerous similarities with that recorded during the actual corresponding physical activity. Thus, mental imagery is a useful neuroimaging strategy that should offer relevant insight into the brain activity underlying non-verbal auditory communication.

In the present study, we conducted an exploratory investigation into the neurocorrelates of auditory communication exchanges between dyads of musicians using MEG hyperscanning. Our primary aim was to identify the cortical oscillatory activities underpinning auditory communication exchange, specifically those related to creative and free exchange. Secondary to this was to understand how these activities might be moderated by social role. To realize this exploration, we used the communication medium of improvisational music, and a musical improvisation turn-taking model to simulate and capture the free creativity and turn-taking dynamics of natural auditory communication. In line with our prior work and numerous other reports in neuroimaging, we targeted brain activity during periods of mental imagery. Additionally in line with our prior work and typical exploratory approaches in encephalographic neuroimaging, we separately analyzed theta (5–7 Hz), alpha (8–12 Hz), and beta (15–29 Hz) source-level activity. We hypothesized that the cortical oscillatory activities which differentiate between natural/free (i.e., improvisational) vs. constrained (i.e., non-improvisational) auditory communication exchange in our two-way hyperscanning paradigm would potentially reveal new neurocorrelates of auditory communication compared to those observed in prior single-subject paradigms and hyperscanning paradigms which focused on brain synchrony. Moreover, we hypothesized that these activities would potentially be moderated depending on the role of each subject during the communication exchange (i.e., which subject was in charge of initiating the communication). Therefore, we used a two-factor whole-head exploratory approach which considered the social role of the participant (leader or follower), and whether communication responses were improvisational (yes or no). This study fills an important gap in hyperscanning-based neuroscience regarding auditory communication and musical improvisation, and is only the second study that we know of to employ musical improvisation performance with MEG.

## Materials and Methods

### Subjects

This study targeted Hokkaido University students who were members of the university’s symphony orchestra. All the subjects knew each other, and were paired according to their scheduling availabilities. Subjects’ playing frequency and frequency of improvisation (both in terms of hours per week) were assessed *via* a music experience questionnaire modeled after that used by Bashwiner et al. (2016). Ten pairs of right-handed musicians (9 males; mean ± SD age, 21.1 ± 1.5 years) with little or no experience with improvisation were selected. There were eight cellists, seven violinists, two percussionists, one violinist, one bassoonist, one oboist, one trombonist, one guitarist, and one drummer (includes those who play more than one instrument). Fifteen subjects practiced no or very little improvisation. Five subjects practiced improvisation on a weekly to daily basis. All subjects could easily perform the tasks in this study. Subjects’ practice frequency with their primary instrument ranged from several times weekly to several hours daily. For further details regarding the characteristics and musical experience of subjects, see Supplementary Table 1. Written informed consent was obtained from all subjects prior to participation in this study, which was approved by the Ethics Committee of the Faculty of Health Sciences and the Ethics Committee of the Graduate School of Medicine, Hokkaido University, and conformed to the 1964 Helsinki declaration and its later amendments or comparable ethical standards.

### Keyboard and Auditory Feedback

We constructed two MEG-compatible keyboards as described in Boasen et al. (2018) for the individual members of a given subject pair to be placed in their respective shielded rooms. Each wooden keyboard had five wooden keys whose depression activated individually placed, circular Piezo sensors. Serial signals from the Piezo sensors were fed outside the keyboard’s respective shielded room into an Arduino circuit board connected to a Windows operated notebook PC. An open-source program was used to convert individual Piezo sensor signals into MIDI. This program was purposely modified to eliminate velocity effects of Piezo sensor activation. In other words, regardless of the strength a key was depressed, the loudness of the sound generated from its activation was uniform across keys. MIDI signals from each key were further programmed to play a major pentatonic scale beginning from the leftmost key with middle C (C3; 261.6 Hz). The pentatonic scale was chosen due to its lack of dissonance, facilitating improvisational expression, potentially even among musically inexperienced subjects whom we hope to target later with this paradigm. Free software was then used to feed these MIDI signals into a virtual MIDI port (Hairless) that was then read by music production software and played through a native MIDI piano instrument plugin [Cakewalk, BandLab Technologies, Version: 2018.09 (Build 29, 64 bit), Singapore]. To provide subjects with auditory feedback of their own performance and simultaneously deliver that performance to the other member of the subject pair, the piano sound output from any given PC was routed through an audio mixer (VR-4HD, Roland) at the PC’s corresponding MEG device site. Sound output from the mixer was split, with one audio stream routed to an electrostatic speaker within the shielded room at the same site, and another stream routed to an identical audio mixer at the opposite MEG site and then routed to an electrostatic speaker within the shielded room there. The latency between Piezo sensor activation and audio output was manually set to 18.7 ms, a time which simultaneously did not burden the processing speed of the PC used, and permitted natural musical performance by the subject. Site-to-site audio latency of the MEG hyperscanning system has been evaluated at 3.13 ms in one direction and 2.78 ms in the other direction, with no jitter (Watanabe et al., 2022). The difference in latency depending on the site is thought to reflect the slight difference in distance of the electrostatic speaker in the shielded room to the device helmet. Regardless, site-to-site latency was considered negligible for the long multi-second epochs used in the present study. Together with the delay of the keyboard sound generation software, the total site-to-site audio latency was less than 22 ms.

### Experimental Design

**FIGURE 1:**
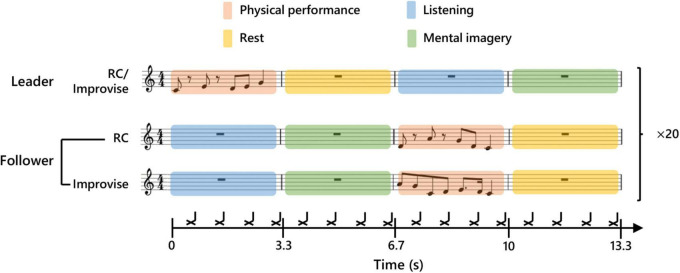
Experimental paradigm. Schematic diagram of communication between Leader and Follower during a single experimental epoch. The upper part of the score shows an example of a Leader’s musical communication. The middle part shows an example of a Follower’s reply during the Rhythm Copy (RC) condition. Twenty consecutive communication epochs were performed for each condition. The lower part shows an example of a Follower’s reply during the Improvise condition. The black cross marks indicate the timing of a backbeat which was used to maintain tempo.

In the present study, subjects participated in pairs in a musical communication task. One subject in a given pair was initially and randomly assigned the role of Leader, while the other subject was assigned the role of Follower. The Leader and Follower communicated to each other musically by playing the keyboard described above using the fingers of the right hand. This musical communication task was modeled after the task used in the single MEG experiment described in Boasen et al. (2018), and resembles a form of musical exchange used in musical improvisation called, “trading”. To facilitate the temporal alignment of musical performance, and ensure temporal regularity in the length of all trials across all subject pairs, a rhythmic backing played throughout the entirety of the musical communication on the off-beat of every eighth note in 4/4 time at a tempo of 72.5 bpm. The tempo was chosen based on extensive pretesting, and represents a compromise between playability, and the need to reduce fatigue and overall experimental time while simultaneously ensuring sufficient trials to average out noise. The first musical communication exchange began after a two-measure intro of the backing rhythm. Then, the Leader commenced the communication by improvising on the keyboard for one measure (0–3.3 s; the Leader’s physical performance period and the Follower’s listening period). In other words, the Leader performed for one measure using any combination and number of notes (i.e., half notes, quarter notes, sixteenth notes, or thirty-second notes) he or she desired, so long as the performance was temporally congruent with the tempo (see Figure 1 for an example). During the measure subsequent to this, the Leader rested, and the Follower mentally imagined her/his own performance response (3.3–6.7 s; the Leader’s rest period and the Follower’s mental imagery period). In the third measure, the Follower was instructed to physically perform what they had mentally imagined in the previous measure (6.7–10.0 s; the Leader’s listening period and the Follower’s physical performance period). In the fourth measure, the Follower rested, and the Leader mentally imagined a new performance to communicate to the Follower (10.0–13.3 s; the Leader’s mental imagery period and the Follower’s rest period). Thus, one musical communication exchange comprised four musical measures, which correspondingly represented one experimental epoch for the present study. A second epoch of musical communication exchange commenced immediately after the first epoch, with no break or disruption to the musical time scale. A continuous series of 20 epochs comprised one communication set. During an experiment, the Leader and Follower performed their musical communication exchange for four sets. After each set, the communication roles were switched. Each subject thus musically communicated as a Leader for two sets, and as a Follower for two sets, during the course of an experiment.

In addition to communication roles, there were also two performance response conditions for the Follower: Improvise, and Rhythm Copy (RC). During the Improvise condition, the Follower was free to respond to the Leader’s musical communication using any combination and number of notes he or she desired, so long as the performance was temporally congruent with one measure of the backing rhythm. During the RC condition, the Follower used any melodic combination of notes to duplicate the rhythmic pattern of the notes performed by the Leader (i.e., the rhythm was copied, but melody was improvised). We purposely avoided using a melodic copy condition, as the level of expertise required to achieve this is beyond that of most subjects, even those with music experience, thereby severely limiting the applicability of this communication paradigm. The performance response conditions were randomly assigned to each of the four communication sets, whereby the Follower performed according to the given response condition for all 20 epochs of that set. Correspondingly, each subject performed as a Follower for one set according to the Improvise response condition, and one set according to the RC response condition, during the course of an experiment. Meanwhile, the Leader musically communicated using rhythmic and melodic improvisation regardless of the performance response condition of the Follower.

The audio for the backing rhythm was created in Cakewalk [BandLab Technologies, Version: 2018.09 (Build 29, 64 bit), Singapore] using an open hi-hat sound in Cakewalk’s default drum kit. The backing rhythm audio was played using Presentation (Neurobehavioral Systems, ver. 19) from a computer at MEG site A. The temporal length of each backing rhythm audio file was one experimental epoch (i.e., four musical measures). Presentation was programmed to send a trigger pulse to the MEG recording at both sites at the start of each backing rhythm audio file (i.e., at the start of each experimental epoch) to facilitate MEG data analysis. As in Boasen et al. (2018) the mental imagery periods were targeted in the analyses of the present study, and this brain activity was normalized according to a two-second period during the rest period (Leader: 4–6 s; and Follower: 11–13 s). The physical performance periods served to keep subjects musically engaged, and permit verification of musical performance behavior (see Behavioral Data Collection). A diagram summarizing this experimental design is shown in Figure 1.

### Behavioral Data Collection

Although the present study expressly focused on brain activity during the mental imagery period, a time when there was no behavioral response, the experiment was designed such that the notes imagined during the mental imagery period are recalled and physically played during the physical performance period. Thus, we assumed that the notes played during the physical performance period were a reasonable representation of the behavioral response during the mental imagery period. The number of notes imagined and correspondingly physically played (hereafter, note count) in each epoch was not controlled in the present study. Therefore, it was important to assess whether note count was affected by the communication roles and/or conditions. Thus, concurrent with MEG recording, keyboard responses during the physical performance period were recorded for each subject in the form of MIDI data. From this MIDI data, mean note counts for each subject for each role and each condition were calculated for use in statistical analyses. Furthermore, to test adherence to the experimental conditions, the difference in note count between the leader and follower was calculated (Follower minus Leader) for each musical communication exchange, and the mean and standard deviation of these differences for each condition in each dyad were calculated. We expected that if followers adhered to the experimental conditions well, the mean and SD for the RC condition should be near or closer to zero than the mean and SD for the Improvise condition. Conversely, we expected the mean and SD for the Improvise condition to be significantly different from zero.

Finally, as a further test of behavioral adherence to the RC condition, we assessed the temporal alignment of successfully copied notes by the Follower with those played by the Leader for each musical communication epoch. This was achieved by importing the MIDI files into MuseScore 3 (Version: 3.0.0, MusicScore BV, Belgium) software, which converts the MIDI into standard musical notation. Leader and Follower exchanges were vertically aligned and visually inspected. For every successfully copied note, the extent of temporal deviation in terms of 16th notes was recorded for each communication epoch. In the case of a missed note, the temporal value of the missing note in 16th notes was recorded. The sum of these 16th note deviations was calculated for each communication epoch and divided by the total number of possible notes to be copied, thus giving the average temporal deviation for any given note the Follower played for that epoch in terms of 16th notes. Then, these values were averaged across all 20 epochs for each subject to produce an average temporal deviation of copied notes in terms of 16th notes. Similarly, for every successfully copied note, the extent of the melodic deviation in terms of position on the keyboard was recorded for each communication epoch to ensure that the RC condition was valid for the dimension of rhythm only and not for melody. As the keyboard was five keys, the maximum melodic deviation was four keys, and the minimum was zero (i.e., melodic duplication). Missed notes were omitted from this analysis. The number of melodic duplications per trial were summed for each trial and divided by the number of copied notes for that trial. This was then averaged across trials for the RC condition for each subject to provide a mean melodic copy rate for the RC condition for each subject. The mean experimental melodic copy rate and standard deviation across subjects are reported. Additionally, the total melodic deviation was summed for each trial and divided by the number of copied notes for that trial. This value was then averaged across trials to provide the mean melodic deviation for the RC condition for each subject. The mean melodic deviation rate and standard deviation across subjects is reported.

### Experimental Procedures

On the day of an experiment, both members of the subject pair were instructed to arrive at the same time at MEG site A where they were greeted by a researcher and were guided to the MEG room. After completing informed consent procedures, subjects were given the Japanese version of the Edinburgh handedness inventory to confirm right handedness. Next, subjects were given a brief tour of the MEG device in the shielded room where the keyboard was installed and ready. They were then provided a hands-on demonstration of how the keyboard functioned. Subjects were then instructed how to perform the experimental tasks and conditions at a desk outside the shielded room, where their comprehension was verified *via* a training session. For the training session, subjects were alternatingly assigned a role of Leader or Follower, and the recorded backbeat was played through desktop PC speakers. The subjects then practiced a series of continuous presentation-response epochs while seated upright in a chair. Then roles were reversed, and they practiced again. Subjects were instructed to assign each finger to just one key on the scale (i.e., the thumb played C3, the index finger played D3, etc.). They were also specifically instructed to not move their heads, trunk, or other extremities, and to move their right hands for performance during the physical performance period only. Once it was clear that subjects understood the instructions and could perform the experiment without difficulty, they were prepared for MEG recording, with one subject remaining at MEG site A, and the other subject guided by a researcher to MEG site B.

Head position indicators, fiducials, and head points were digitized according to standard MEG operating procedure (Hansen et al., 2010). The subject was positioned in an upright position in the MEG measurement chair, onto which a table was attached. Upon the table, the MEG keyboard was fixed with tape at a comfortable position for right-handed performance. The subjects then placed their right hand in position on the keyboard, with their palm resting on the table, and each fingertip resting on the surface of its corresponding key. A box was then placed over top of the keyboard and their right hand to prevent visual distraction from hand and finger movement. Subjects were further instructed to look straight ahead at a fixation cross which was drawn on a piece of paper and taped at eye level on the inner wall of the shielded room. Subjects were also re-instructed to move only their right hand, and only during the physical performance period. At all other times, they were instructed to rest the fingers of their right hand on the surface of the keys, and their right palm on the surface of the table. Keyboard output volume at both sites was adjusted using the audio mixers such that it was comfortable for both subjects. Prior to the start of each experimental communication set, the experimenter used an external microphone connected to the electrostatic speaker in the shielded room to inform subjects about the relevant forthcoming presentation-response roles and condition, and to verify that the subjects understood their roles and how to respond appropriately. Throughout the experiment, subjects were visually and aurally monitored to ensure comfort and compliance with all experimental instructions.

### Magnetoencephalography Recording and Processing

The present study was conducted using the MEG hyperscanning system at Hokkaido University which comprises a 101 ch custom Elekta-Neuromag MEG and a 306 ch Elekta-Neuromag Vector View MEG, directly connected *via* fiber optic cables. The system is synchronized *via* trigger transmission from the stimulus presentation computer at MEG site A. One-way trigger latency of the MEG hyperscanning system has been evaluated at 1.8–3.9 μs (Yokosawa et al., 2019). All MEG measurements were done within magnetically shielded rooms.

Magnetoencephalography signals were online band-pass filtered from 0.6 to 200 Hz and recorded at a 600 Hz sampling frequency. All MEG data processing was performed in Brainstorm^1^ [run on MATLAB 2019b (MathWorks, Natick, MA, United States)]. This processing began with removal of noisy or dead channels (mean = 6.8 channels/subject). Physiological artifacts related to eye blinks, heartbeat and/or periodic noise related to signal packet transmission from the MEG device to the recording computer were isolated and removed using independent component analysis. Components for removal were identified *via* visual inspection of both their topographical signal pattern and their time-series waveform. On average 0.7 ± 0.6 (mean ± SD) components were removed per subject. A comb filter was applied at 50 Hz and related harmonic frequencies to remove line noise. A band-pass filter was then applied from 1 to 40 Hz. Cleaned and filtered data was then epoched at −1 to 14.3 s relative to each physical performance period of the Leader. Each epoch was visually scanned, and those with noise spikes exceeding 1,000 fT in amplitude (presumed to be movement artifacts) were removed. Subject head points and fiducials were coregistered to a common template brain (ICBM152). An overlapping-sphere forward model was computed, and minimum-norm estimation was used to calculate cortical currents without dipole orientation constraints at 15,002 voxels. The time-series of the cortical currents at each voxel was decomposed into the theta (5–7 Hz), alpha (8–13 Hz), and beta (15–29 Hz) frequency bands, and their corresponding envelopes computed using Hilbert transform. Time-frequency envelopes at each voxel in each frequency band were averaged across epochs within subjects for each condition. Subsequently, the amplitude of the time-frequency envelopes was standardized across subjects as a percent deviation from baseline using the following equation where x is the amplitude of the time-frequency envelope at each time point, and μ is the time-average over the baseline period (see Figure 1).


$$X_{std}=\frac{x-\mu }{\mu }\times 100$$

Standardized cortical time-frequency envelopes were averaged over the mental imagery period for each frequency band for each subject for each role and condition separately. The resulting maps of cortical time-frequency activity were exported as GIFTI surface data files for use in statistical analyses.

### Statistical Analyses

Despite that Leaders were instructed to improvise, regardless of the playing condition of the Follower, we cannot rule out that the playing condition of the Follower somehow influenced the behavior and/or brain activity of the Leader. Indeed, it is precisely the goal of this study to explore the possibility of such an interaction. Therefore, we opted for a two-by-two framework for our statistical analyses.

To assess how experimental roles and conditions affected musical communication behavior, mean note counts and mean rhythm variance scores were separately analyzed using a two-way repeated measures analysis of variance (RM ANOVA) [(role: Leader, Follower) × (condition: RC, Improvise)]. Furthermore, to test adherence to the experimental conditions, the mean and standard deviation of the difference in note counts between leaders and followers were compared between conditions and against zero using a paired *t*-test and single sample *t*-test, respectively. These tests were performed using SPSS 26 (IBM), with significance determined at *p* ≤ 0.05.

To explore how cortical activity was affected by communication roles and conditions, mean standardized cortical activity over the mental imagery period was analyzed separately for each frequency band of interest using SPM12 [v7771, run on MATLAB 2019b (MathWorks, Natick, MA, United States)] by constructing a two-way within-subjects flexible-factorial model [(role: Leader, Follower) × (condition: RC, Improvise)], and then estimating a classical inference model. Then, F contrasts for the main factors of role and condition and the interactions between main factors were mapped for each frequency band separately using a height threshold of *p* = 0.05 (uncorrected), with significant clusters determined based on a cluster-defining threshold of *p* < 0.001, followed by a cluster-wise FWE corrected threshold of *p* < 0.05, with a minimum cluster extent of 50 voxels. In the case of significant clusters, brain activity in the cluster was extracted to clarify differences within/between factors. *Post hoc* single-sample *t*-tests were also conducted to further assess if the involved activity during the mental imagery period significantly deviated from baseline levels.

## Results

### Behavioral

**FIGURE 2:**
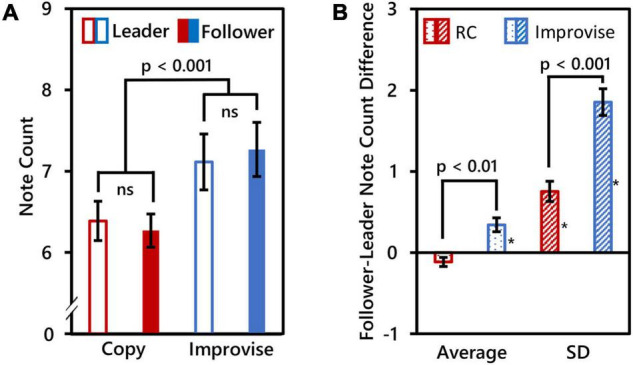
Behavioral comparisons. **(A)** Mean note counts for Rhythm Copy (RC) and Improvise in the Leader and Follower roles (*N* = 17). Results showed that mean note counts in the Improvise conditions were significantly higher than that in the RC conditions, regardless of the roles of Leader and Follower. This indicated an increase in the degree of freedom of the rhythm in the Improvise conditions. **(B)** Average and standard deviation (SD) of Follower minus Leader note counts across epochs (*N* = 14), indicating a significant increase in note count variation between Followers and Leaders in the Improvise condition. Asterisk indicates significant differences from 0 based on single-sample *t*-tests.

Due to technical issues, MIDI recordings for three subjects were unsuccessful. Therefore, behavioral within-subjects comparisons are based on 17 subjects, and between-subjects comparisons are based on seven pairs of subjects (i.e., *N* = 14). The number of notes played by Leaders was 6.388 ± 0.996 (mean ± SD) for the RC condition, and 7.115 ± 1.416 (mean ± SD) for the Improvise condition. The number of notes played by Followers was 6.271 ± 0.844 (mean ± SD) for the RC condition, and 7.268 ± 1.372 (mean ± SD) for the Improvise condition. RM ANOVA revealed no significant interaction in note count between role and condition (*F*_(1_,_16)_ = 0.360, *p* = 0.557), nor significant difference in note count between roles (*F*_(1_,_16)_ = 0.010, *p* = 0.923). However, there was a significant difference in note count between conditions (*F*_(1_,_16)_ = 55.170, *p* < 0.001), with more notes played for the Improvise condition compared to the RC condition regardless of roles (mean ± SE: 7.191 ± 0.329 vs. 6.329 ± 0.218, respectively). See Figure 2A for a visual summary of these results.

Paired *t*-test results comparing mean and SD of the Follower minus Leader note count differences between the Improvise and RC conditions revealed significant differences for both the mean [0.343 ± 0.0864 vs. −0.114 ± 0.056, respectively (mean ± SE); *p* = 0.006] and SD [1.854 ± 0.166 vs. 0.754 ± 0.124, respectively (mean ± SE); *p* < 0.001], thus indicating significantly greater variance in the number of notes played between Leaders and Followers during the Improvise condition compared to the RC condition. Single-sample *t*-tests revealed that the mean Follower–Leader difference in note count was not significantly different from zero in the RC condition (*p* = 0.088), but was significantly greater than zero in the Improvise condition (*p* = 0.007), thereby indicating that Followers significantly varied their responses to leaders when improvising. The mean of the SD of the follower-leader note count difference was significantly different from zero in both the RC and Improvise conditions (*p* = 0.001, and *p* < 0.001, respectively), supporting not only the increased variance in note count observed in the Improvise condition, but also indicating that, despite the general adherence to the RC condition, Followers were not perfect at copying what Leaders played. Interestingly, the mean Follower minus Leader note count difference for the RC condition was negative, indicating that when Followers made errors during the RC condition, they tended to do so by playing less notes. Conversely, the mean Follower minus Leader note count difference for the Improvise condition was positive, indicating that when Followers responded to Leaders during improvisation, they tended to do so by playing more notes. See Figure 2B for a visual summary of these results.

Finally, as per our analysis of temporal alignment of copied notes, for any given note played by Leaders, Followers were on average 0.51 16th notes off on the timing of their copied note with a standard deviation of ±0.17 16th notes. This means that the participants in this study were never more than a 16th note off on the timing of their play during the RC condition, and usually only a 32nd note off, which is a very small deviation. Importantly, we should remind readers that this temporal deviation result includes a time penalty for missed notes. If the analysis had only considered successfully copied notes, the mean temporal deviation would have certainly been less than a 32nd note, which is highly accurate. Indeed, aside from when notes were missed, aural monitoring of the communication of subject pairs during the experiments indicated clear and recognizable mimicry by Followers of the rhythm played by the Leaders. As to melodic copy rate, the melody of rhythmically copied notes was the same as that played by Leaders at a rate 33.4 ± 18% (mean ± SD), meaning that both the rhythm and the melody were copied for approximately one note out of every three notes played by the Follower. However, the rate of melodic deviation was 1.3 ± 0.41 key steps (mean ± SD) for every note played, meaning that despite some melodic duplication, Followers were consistently modifying the melodic pattern of their response. This phenomenon was clearly audible and noted for all subjects throughout this study. Thus, we conclude that from a behavioral standpoint, the task was well understood by the subjects and executed successfully.

### Theta (5–7 Hz)

**FIGURE 3:**
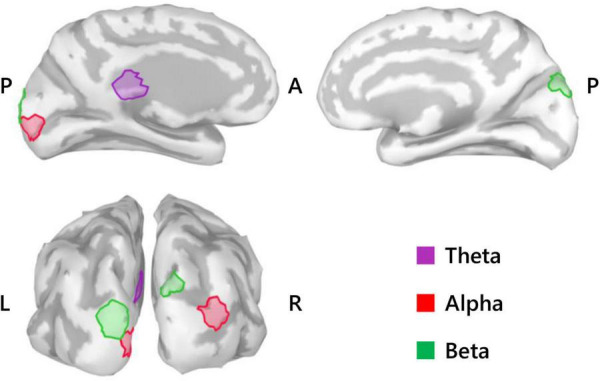
Summary of SPM results (*N* = 20). Medial view (top) and posterior view (bottom left) cortical maps highlight the significant clusters (50 voxels) for the theta activity interaction between role and condition, the alpha activity main effect of role, and the beta activity main effect of role. L, left; R, right; A, anterior; P, posterior.

**FIGURE 4:**
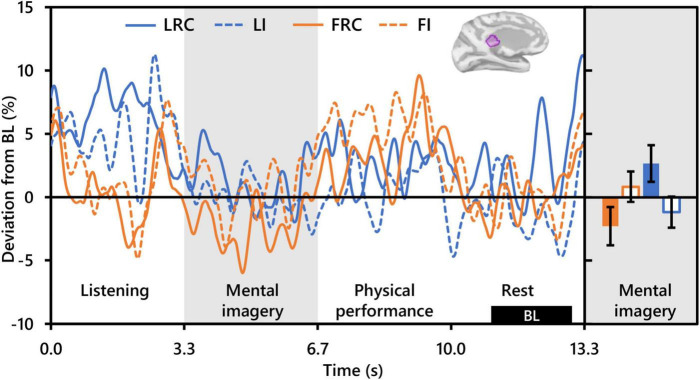
Interaction between role and playing condition with theta activity (*N* = 20). The time-series waveforms (left) show overall mean theta (5–7 Hz) activity in the left significant cluster in the isthmus cingulate (see displayed cortical map) across one communication epoch for the Rhythm Copy (RC) and Improvise conditions for Leaders and Followers separately and temporally aligned to facilitate visual comparison. The histogram (right) shows representative overall mean activity over the mental imagery period in the same cluster for each role/condition. LRC, leader rhythm copy; LI, leader improvise; FRC, follower rhythm copy; FI, follower improvise; BL, baseline period. Error bars are standard error.

Repeated measures analysis of variance of theta activity during mental imagery revealed no significant clusters for the main effect contrast of role, nor any for that of condition. However, the contrast for the interaction between role and condition revealed a significant cluster situated in the left isthmus cingulate cortex (ICC) (P_*FWEcorr*_ = 0.033; MNI: −1, −42, 17) (see Figure 3). Representative mean time-series theta activity in this cluster for each role and playing condition (Figure 4, left) reveals a complex interplay between role and condition, with differences seemingly favoring a dichotomy between social role during listening which diverges into an interaction state during the mental imagery period. As can be discerned by the histogram on the right side of Figure 4, theta activity during mental imagery is higher than baseline in Leaders and lower than baseline in Followers during the RC condition. However, this relationship trends in the opposite direction during the Improvise condition. None of these deviations from baseline reached statistical significance in *post hoc* single sample *t*-tests.

### Alpha (8–13 Hz)

**FIGURE 5:**
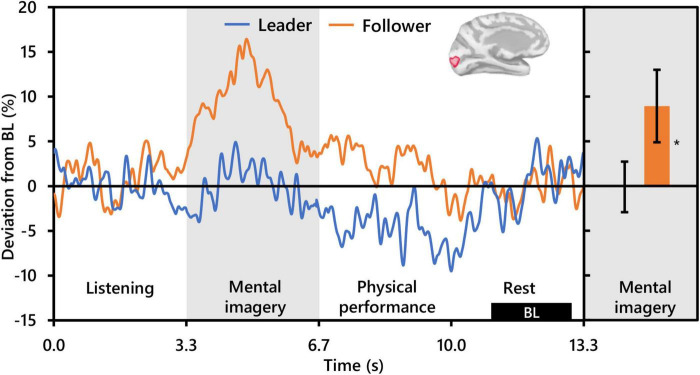
Effect of social role on occipital alpha activity (*N* = 20). The time-series waveforms (left) show overall mean alpha (8–13 Hz) activity in the left significant cluster which overlapped portions of the cuneus, pericalcarine, lingual gyrus, and lateral occipital areas (see displayed cortical map) across one communication epoch for Leaders and Followers separately and temporally aligned to facilitate visual comparison. The histogram (right) shows representative overall mean activity over the mental imagery period in the same cluster for each role. BL, baseline period. Error bars are standard error. Asterisk indicates significant differences from 0 based on single-sample *t*-tests.

With respect to alpha activity, contrasts for the main effect of condition and the interaction between condition and role revealed no significant clusters. However, the contrast for role revealed two significant clusters bilaterally in the occipital cortex. The cluster in the left hemisphere was centered at the intersection of the pericalcarine cortex (PeC), the cuneus cortex (Cu), and the lateral occipital cortex (LOC) (P_*FWEcorr*_ = 0.002, MNI: −1, −95, −3), and that in the right hemisphere was centered at the intersection of the LOC and the inferior parietal cortex (IPC) (P_*FWEcorr*_ = 0.004, MNI: 41, −88, 14) (see Figure 3). Visually comparing representative mean time-series of alpha activity in the left occipital cluster for each social role separately (Figure 5, left), one can see pronounced synchronization in Followers commencing from the start of the mental imagery period until the midpoint. Conversely, alpha activity in Leaders exhibits slight desynchronization just prior to the commencement of the mental imagery period. Once mental imagery begins, Leaders exhibit mild alpha synchronization until the midpoint. From the midpoint onward, alpha activity in both Leaders and Followers begins to desynchronize. The resulting net change in alpha activity in Leaders during mental imagery is not much different than baseline levels, but significantly elevated in Followers (Figure 5, right). The desynchronization that both Leaders and Followers exhibit prior to physical performance is a typical response pattern associated with motor preparation (Caetano et al., 2007) which was likewise observed in our previous use of this music performance paradigm (Boasen et al., 2018). Desynchronization in both Leaders and Followers appears to culminate at the same timepoint just at the end of physical performance. Notably however, while alpha activity in Leaders desynchronizes below baseline levels during physical performance, it remains above baseline in Followers from the start of mental imagery all the way through the end of physical performance.

### Beta (15–29 Hz)

**FIGURE 6:**
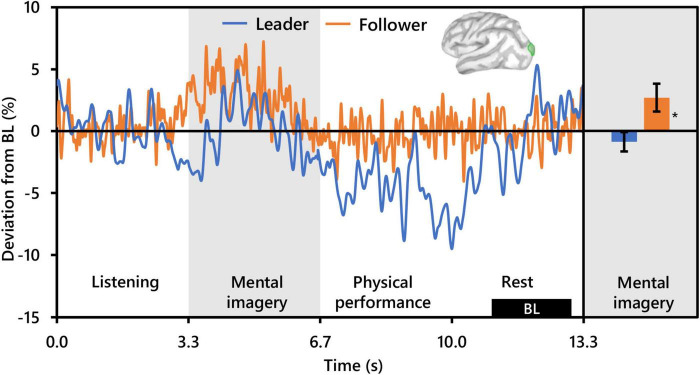
Effect of social role on occipital beta activity (*N* = 20). The time-series waveforms (left) show overall mean beta (15–29 Hz) activity in the left significant cluster overlapping the lateral occipital cortex (see displayed cortical map) across one communication epoch for Leaders and Followers separately and temporally aligned to facilitate visual comparison. The histogram (right) shows representative overall mean activity over the mental imagery period in the same cluster for each role. BL, baseline period. Error bars are standard error. Asterisk indicates significant differences from 0 based on single-sample *t*-tests.

Regarding beta activity, contrasts for the main effect of condition and the interaction between condition and role revealed no significant clusters. However, the beta contrast for role revealed two significant clusters bilaterally in the occipitoparietal cortex, similar but slightly more superiorly centered compared to those revealed by the alpha contrast for role. The cluster in the left hemisphere was centered in the superior portion of the LOC (P_*FWEcorr*_ < 0.001, MNI: −13, −103, 18), and that in the right hemisphere was centered at the intersection of the LOC and the superior parietal cortex (SPC) (P_*FWEcorr*_ = 0.001, MNI: 12, −90, 42) (see Figure 3). Visually comparing representative mean time-series beta activity in the left significant cluster for each social role separately (Figure 6, left), one can see response dynamics similar to those which were observed in the alpha band. Indeed, the higher beta activity in Followers appears to be driven by a synchronization event in Followers commencing from just prior to the start of the mental imagery period until the midpoint. Again, this synchronization event appears in Leaders, but starts later and is more subdued. Then, from the midpoint onward, beta activity in both Leaders and Followers begins to desynchronize, likely in association with motor preparation (Caetano et al., 2007). However, beta activity in Leaders drops well below baseline prior to and throughout physical performance, whereas beta activity in Followers returns to baseline levels at the start of physical performance and remains there until the end of the communication epoch. Net changes in beta activity during the mental imagery period were not different from baseline in Leaders, but significantly above baseline in Followers (Figure 6, right).

## Discussion

The present study aimed to identify the neural oscillatory underpinnings of auditory communication exchange *via* musical improvisation between dyads of classically trained musicians using MEG hyperscanning. We compared the differences in brain activity during mental imagery of musical performance according to whether rhythm was copied or improvised (playing condition), and whether the dyad member was a Leader or a Follower in the communication (social role). Behaviorally, all dyad members appeared to perform the playing conditions and social roles well. Analyses of brain activity revealed a significant interaction between playing condition and social role in the theta band, and significant effects of social role in the alpha and beta bands, with the involved brain areas differing according to frequency band. To our knowledge, this is the first MEG hyperscanning study to investigate the neurocorrelates of not only two-way auditory communication, but also two-way improvisational music performance.

Behavior analyses showed that the number of notes was significantly higher in the Improvise condition than in the RC condition regardless of social role (Figure 2A). A similar result was observed in our previous work (Boasen et al., 2018). Given that the time frame of the musical measure for performance is fixed, increased note count implies greater rhythmic complexity. This is interesting, as there is no intrinsic reason why the RC condition could not have been as rhythmically complex because Leaders were free to play whatever they wanted. Although we did not conduct post-experiment interviews to confirm it, we speculate that Leaders may have purposely tried to limit the rhythmic complexity of their communication during the RC condition to ensure that Followers could copy the rhythm correctly. Indeed, the phenomenon of humans constraining or attuning aspects of their communication in accordance with the situation or the skill of the person with whom they are communicating with is well-known in social science (Francik and Clark, 1985; Fowler and Levy, 1995; Krauss and Chiu, 1998). In the Improvise condition, this conscious constraint was apparently lifted, and Leaders played more freely. Followers likewise increased the rhythmic complexity of their playing while significantly varying their response from Leaders as they were supposed to in the Improvise condition (Figure 2B). Thus, both playing condition and social role influenced musical communication behavior, and underscore the differences in cognitive brain activity we have observed.

Of the three frequency bands analyzed, only theta band activity was useful for differentiating playing condition, although this effect significantly interacted with that of social role. The spatial location of this differential activity was localized only in the left hemisphere in an area overlapping the isthmus of the cingulate cortex (ICC). Considered a key hub and mediator in the default mode network and passive internal processing (Sandhu et al., 2021), the ICC links the thalamus to other cortical areas, and damage to has been observed to affect verbal and visual memory and visuospatial processing (Valenstein et al., 1987; Katayama et al., 1999; Maeshima et al., 2001). Relevance of the ICC to improvisational music performance has not been highlighted in past studies (Limb and Braun, 2008; de Manzano and Ullén, 2012; Donnay et al., 2014). Nor was activity in the ICC observe to differentiate between rhythmically constrained and free improvisation during performance imagery in our prior work with single subjects (Boasen et al., 2018). Moreover, the ICC has not been noted as a region of interest in hyperscanning studies on auditory communication (Kelsen et al., 2022). However, in a study of musical imagery, Bastepe-Gray et al. (2020) observed increased activation in the nearby posterior cingulate cortex (although right lateralized) when subjects visualized their hands playing piano compared to eyes closed rest. Interestingly, reduced cortical thickness in the ICC has been related to higher imagination and creative achievement scores in healthy adults (Jung et al., 2016; Wertz et al., 2020), something the authors attributed to neural processing efficiency and synaptic pruning, an idea supported by other studies on creative ability (Tian et al., 2018; Vartanian et al., 2018).

Despite the lack of a clear role of the ICC in improvisational musical performance or interpersonal aspects of communication targeted by other hyperscanning studies, its relationship with visual, verbal, and spatial processing and creative ability supports a possible role for the ICC in creative auditory communication ideation. Indeed, the ICC has been implicated as an important hub in decision making when processing the acoustic properties of speech (Mahmud et al., 2021). Supposing ICC theta activity to potentially reflect the extent of creative auditory communication ideation, a path to interpreting the significant interaction between playing conditions and social roles opens. The lowest level of ICC theta activity was exhibited by Followers during the RC condition, which should have involved the least amount of creative ideation out of the four condition/role combinations. Conversely, during the Improvise condition, Followers exhibited higher ICC theta activity which trended above baseline. Paradoxically however, the ICC theta activity of Leaders during the Improvise condition, although it trended above Followers during the RC condition, trended lower than Followers during the Improvise condition. The Leader/Improvise combination should have permitted the freest communication ideation of all four combinations. However, therein may lie our explanation. During the Improvise condition, Leaders did not need to consider the response from the other dyad member during ideation, something that Followers were instructed to do during Improvise, and something that Leaders spontaneously did during RC. Indeed, our behavioral results support that Leaders were making a conscious effort to reduce the complexity of their communication (see Figure 2), likely out of consideration of the Follower and the Follower’s ability to duplicate the rhythm of the communication. The internal deliberation associated with this phenomenon may therefore explain why ICC theta activity tended to be highest for Leaders during the RC condition. Altogether, the present results indicate a role for the ICC in the cognitive processing underlying auditory communication exchange, which is simultaneously moderated by social role.

This last observation is punctuated by our alpha activity results which indicated a significant effect of social role. However, contrary to Konvalinka et al. (2014), who observed differential alpha activity due to social role in the prefrontal cortex, social role in the present study was differentiated by alpha activity bilaterally in the occipital cortex. Specifically, Followers exhibited robust synchronization that was absent in Leaders from the start to the midpoint of mental imagery resulting in significantly higher mean alpha levels than Leaders during mental imagery in clusters centered in the right hemisphere at the intersection of the LOC and the IPC and in the left hemisphere at the intersection of the PeC, Cu, and LOC (see Figure 3). The areas coincide with the visual cortex (Zeki et al., 1991), and are important for not only visual processing but also the spatial, contextual, and categorical representation of sound (Petro et al., 2017; Campus et al., 2019; Mattioni et al., 2020). Importantly, when contrasting with baseline conditions, functional activation of the occipital cortex in musicians during musical performance (Limb and Braun, 2008; Donnay et al., 2014) and during musical performance imagery has been observed (Meister et al., 2004; Bastepe-Gray et al., 2020). Meanwhile, recall that the mental imagery performed in the present study involved improvisation, or spontaneous creative ideation of novel melodies, regardless of condition. Creative ideation is thought to be facilitated by bottom-up processing and associated with alpha synchronization (Fink and Benedek, 2014; Adhikari et al., 2016; Lopata et al., 2017), although in the case of Adhikari et al. (2016) alpha synchronization was stronger for pre-learned compared to improvisational music. The latter point aside, the above evidence might suggest that Followers exhibited more robust creativity during the imagery of their performance compared to Leaders. However, there is no plausible task-based reason why this should be. If anything, Leaders should have exhibited brain activity more indicative of creative ideation than Followers, as Leaders were required to improvise not only melody but also rhythm for both playing conditions. Moreover, this interpretation does not sufficiently address the fact that alpha synchronization during creative ideation tends to be localized in frontal-parietal brain areas (Fink and Benedek, 2014), and that musical imagery is generally observed to additionally recruit auditory, language and motor related brain areas (Zhang et al., 2017; Gelding et al., 2019). Another possible explanation is increased active inhibition of task irrelevant visual stimuli, a common interpretation of occipital alpha synchronization in visual tasks (Kelly et al., 2006). Actively inhibiting the input of irrelevant visual stimuli in order to focus on musical response imagery is certainly a plausible cognitive activity that subjects could have been undertaken during the present task. However, again, occipital alpha synchronization was not observed in Leaders (see Figure 4), and there is no task-based reason why Leaders would not have engaged in the same level of inhibition as Followers. Thus, it seems likely that there is another cognitive function in addition to musical imagery that underlies the differential alpha activity patterns between social roles. We propose this cognitive function to be working memory retention.

The relationship between alpha-band activity and the occipital cortex is well-known in working memory research (Sternberg, 1966). Although the observed similarities between social roles with respect to note counts offer no reason to suspect that the processing load related to working memory encoding of imagined information during mental imagery should be any different between social roles, that does not discount the possibility of differences in working memory retention of communicated information from the other dyad member. Indeed, working memory retention is regarded as a key function in auditory communication that permits the listener to hold incoming auditory information while deciphering its semantic and episodic relevance (Lemke and Besser, 2016). Event-related alpha synchronization distributed across parietal-occipital sensors (Wianda and Ross, 2019) and brain areas (Takase et al., 2019) is a typical characteristic observed during visual memory retention, with the extent of synchronization linked to memory load (Hu et al., 2019; Proskovec et al., 2019). Compared to visual memory, publications based on auditory memory are scant. However, Kaiser et al. (2007) similarly observed alpha synchronization diffusely across parietal-occipital MEG sensors during the memory retention period of a short-term auditory spatial memory task. Likewise, Krause et al. (1996), who studied auditory working memory by presenting sequences of vowel sounds to subjects in an auditory-based version of the Steinberg task, observed significant synchronization of alpha activity associated with memory retention widely across parietal-occipital EEG electrodes. Notably, similar to visual serial working memory tasks, the significant alpha synchronization in the Krause et al. (1996) study began roughly at the end of memory item presentation and lasted until the presentation of a cue to commence memory recall. The auditory communication in the present study has parallels to serial working memory tasks in the sense that the auditory rhythm and melody information is transmitted in series from one dyad member to another during the listening/physical performance period, and that the start of mental imagery period coincides exactly with the completion of this auditory information transmission in each epoch. Certainly during the RC condition, Followers needed to remember the rhythm of the communication from Leaders while imagining their own unique melodic response that retained the rhythm played by the Leader. Thus, memory retention-related occipital alpha synchronization in Followers during mental imagery in the RC condition is logical. However, the marked alpha synchronization in Followers during mental imagery was not condition specific. This suggests that increased memory retention of communication information from Leaders was a general phenomenon attributable to the social role of being a Follower. One could furthermore speculate that this retained information influenced the response imagery of Followers.

Support that the perceived social hierarchical status of a speaker can mediate memory retention of speech information has been demonstrated by Holtgraves et al. (1989), who observed that subjects who read a conversation between two speakers rated as high and low social status had higher memory retention for the utterances of the high-status speaker compared to those of the low status speaker. Their results suggest that humans may intrinsically pay more attention to communication information originating from higher status individuals. This notion is corroborated by Foulsham et al. (2010), who showed that when watching videos of people involved in group decision-making, observers will fixate more frequently and for longer duration on people in the video who had been rated as having higher social status. Translating these ideas to the present study, it is possible that subjects considered the Leader role as higher in hierarchy than the Follower role. Correspondingly, Followers may have paid more attention to Leader communication than the inverse, thereby resulting in more robust retention of Leader communication in Followers as they imagined their response. Conversely, aside from condition-based concerns over the complexity of their playing, Leaders may have communicated in a manner that was largely independent of Follower response, resulting in little need for memory retention, and thereby resulting in the observed occipital alpha activity during mental imagery that did not deviate markedly from baseline levels (see Figure 5, right). Here it is worth noting that that the analytical comparison between social roles in the present study was within subjects, which suggests that the brain activity of an interlocutor during communication can be altered through arbitrary assignment of a hierarchical communication role.

The present beta results are congruent with this interpretation. Beta activity levels were significantly higher in Followers than Leaders bilaterally in occipital clusters centered in the right hemisphere at the intersection of the Cu and SPC, and in the left hemisphere at the intersection of the LOC and SPC. These difference appeared to be driven by pronounced synchronization in Followers, which was present but subdued in Leaders, from the start of mental imagery until the midpoint (see Figure 5). Parietal-occipital beta synchronization is commonly observed during both visual and auditory working memory retention (see reviews by Wilsch and Obleser, 2016, and Pavlov and Kotchoubey, 2020), commencing in parallel to the onset of alpha-band synchronization (Proskovec et al., 2019), and with amplitude increasing as a function of load (Michels et al., 2010; Obleser et al., 2012; Kottlow et al., 2015). Thus, from an oscillatory activity perspective, the present beta results offer further corroboration of greater engagement of working memory retention processing in Followers compared to Leaders during mental imagery. However, from a spatio-functional perspective, evidence of Cu involvement during auditory working memory retention is scant, with more rostral-ventral areas of the precuneus or the cerebellum more commonly reported (Grasby et al., 1994; Kumar et al., 2016). The Cu is structurally are part of the primary visual cortex (Dehaene and Cohen, 2011), and indeed significant activation in the Cu and PeC, along with the LOC has been reported during visual working memory retention (Lepsien et al., 2011; Rämä et al., 2001). Moreover, increased gray matter of the right Cu and left LOC and PeC has been reported in association with increased visual working memory performance (Owens et al., 2018). Together, this evidence supports the beta-oscillatory functional and structural relevance of these areas to working memory processing while also implicating involvement of the visual system.

That the visual system would be involved during musical imagery in musicians is not surprising. Visualization of the actual act of playing one’s instrument, or kinesthetic imagery, is one of the fundamental ways in which musicians imagine playing music (Lotze, 2013), and as previously mentioned, music performance and music performance imagery has frequently been shown to involve areas of the visual system (Donnay et al., 2014; Bastepe-Gray et al., 2020). Although we did not confirm it, we suspect that the musicians in the present study may have used kinesthetic imagery not only to imagine their musical communication during the mental imagery period, but also to listen to and retain the communication from the other dyad member, as the instrument and sounds generated for both members were identical. This may well explain why, despite there being no visual stimulus used in the present study, spectro-spatial characteristics highly indicative both of working memory retention and visual processing were relevant for differentiating between social roles. Regardless, the striking difference in alpha and beta activity characteristics between Followers and Leaders in the present study strongly indicates that the cognitive strategies employed by the subjects differed according to the social role that they were assigned. Thus, the present results offer convincing evidence of the need to control for social role/hierarchy in both the design and analytical models of musical and social neuroscience research.

Altogether, the approach of the present study to investigate intra-brain cortical oscillatory drivers of natural/free auditory communication exchange represents a departure from the typical targets of brain synchrony reported in past hyperscanning studies on auditory communication. In line with our hypotheses, the neurocorrelates we have identified are novel, and were moderated by social role. Overall, the findings indicate that there is more to auditory communication than just interpersonal synchrony, and that more work is needed to bridge the neurocorrelates of inter-brain synchrony during auditory communication with the intra-brain neurocorrelates that drive auditory communication ideation.

The present study had some limitations that are worth acknowledging. First, the subjects targeted in the present study were classically trained musicians with little to no habitual improvisational practice experience. The lack of differential prefrontal activity related to playing conditions may reflect this, and the extent to which the lack of improvisational music experience among the present subjects influenced the differences in social roles we observed remains to be clarified. Second, behavioral differences according to playing condition and social roles, even when considering melody, were primarily assessed based on the rhythmic structure of the communication. The primary reason for this is that melody is arguably a much more complicated musical variable to assess. However, it is possible that behavioral support for our interpretation that Follower responses were dependent upon Leader communication could be identified *via* a thorough exploration of melody. Nevertheless, analyses of note count were sufficient to confirm adherence to playing conditions. Moreover, note count analyses also revealed some dependence of Follower response on Leader communication based on the increased note count of Follower communication in correspondence with Leaders during the Improvise condition. Third, because the present design featured melodic improvisation in both playing conditions, our insight into the cognitive underpinnings of natural/free auditory communication was primarily through the lens of rhythmic improvisation. Future implementations of the present paradigm should perhaps consider implementation of a RC condition where both rhythm and melody are constrained. Fourth, the present design requires subjects to engage memory function during the imagery period so that they can play back their imagined communication. Although we believe the effect of this memory engagement to have been similar across conditions/social roles, and therefore should have canceled out in our statistical comparisons, the underlying cognition is not necessarily the same as in other study designs without a memory requirement and could potentially contributed to the novelty of our results. Finally, post-experiment interviews were not conducted which could have strengthened our interpretations, particularly regarding the cognitive strategies used according to social role. Nevertheless, this shortcoming should not detract from the novel and important finding that brain area-specific alpha and beta activities were different, effectively implying in and of themselves the emergence of different cognitive strategies according to the assignment of social role during auditory communication.

## Conclusion

The present study is the first MEG hyperscanning study to investigate and identify neuro oscillatory drivers of non-verbal auditory communication exchange between two people *via* music performance. Using a paradigm based on musical communication and adapted from musical neuroscience, the present study identified differential spectro-spatial brain activities during mental imagery of music performance according to performance condition and the social role assigned to the subjects. Increased theta activity was observed in the left ICC, potentially in association with the extent of internal deliberation involved in the auditory communication ideation. Meanwhile occipital alpha and beta synchronization indicative of working memory retention processing in coordination with the visual system was observed, regardless of playing condition, in subjects when assigned the roll of Follower but not in the same subjects when assigned the role of Leader. The results offer compelling evidence for both musical and social neuroscience that the cognitive strategies, and correspondingly the memory and attention-associated oscillatory brain activities of interlocutors during communication differs according to their social role/hierarchy, thereby indicating that social role/hierarchy needs to be controlled for in social neuroscience research. Future MEG hyperscanning studies should investigate brain connectivity patterns which underly the present results, in addition to exploring the intra-brain neurocorrelates associated with other interactive communication paradigms.

## Data Availability Statement

The raw data supporting the conclusions of this article will be made available by the authors, without undue reservation.

## Ethics Statement

The studies involving human participants were reviewed and approved by the Ethics Committee of the Graduate School of Medicine, Hokkaido University. The patients/participants provided their written informed consent to participate in this study.

## Author Contributions

JB conceived the study design and built the musical instruments. NY, HW, AS, and KT executed the experiments and collected the data. NY performed the data analyses with guidance from JB and advice from HW, AS, KT, TS, KaY, HS, and KoY. NY and JB wrote the manuscript. JB, TS, and KoY acquired the funding. KoY managed the funding. All authors contributed to the editing and revision of the final manuscript.

## Conflict of Interest

The authors declare that the research was conducted in the absence of any commercial or financial relationships that could be construed as a potential conflict of interest.

## Publisher’s Note

All claims expressed in this article are solely those of the authors and do not necessarily represent those of their affiliated organizations, or those of the publisher, the editors and the reviewers. Any product that may be evaluated in this article, or claim that may be made by its manufacturer, is not guaranteed or endorsed by the publisher.

## Footnotes

1
http://neuroimage.usc.edu/brainstorm
